# Mollifying green skepticism: Effective strategies for inspiring green participation in the hospitality industry

**DOI:** 10.3389/fpsyg.2023.1176863

**Published:** 2023-06-15

**Authors:** Eunjin (Anna) Kim, Eunseon Kwon, Seoyeon Hong, Heather Shoenberger, Marla Royne Stafford

**Affiliations:** ^1^Annenberg School for Communication and Journalism, University of Southern California, Los Angeles, CA, United States; ^2^Bob Schieffer School of Communication, Texas Christian University, Fort Worth, TX, United States; ^3^Edelman College of Communication and Creative Arts, Rowan University, Glassboro, NJ, United States; ^4^Donald P. Bellisario College of Communication, The Pennsylvania State University (PSU), University Park, PA, United States; ^5^Less Business School, University of Nevada, Las Vegas, NV, United States

**Keywords:** narrative message, two-sided message, message usefulness, skepticism, green participation

## Abstract

Environmental awareness is a growing concern for consumers, and effective green messaging strategies are crucial for businesses. This 2 × 2 between-subject experiment investigates the influence of message style and sidedness on consumer participation in green practices and explores the role of message usefulness and skepticism. Our results show that a narrative message style and a two-sided message increase perceived usefulness, reduce skepticism, and lead to greater behavioral intent. Further, the study supports the moderated serial mediation role of message usefulness and skepticism. These findings offer significant implications for businesses seeking to promote sustainable practices and engage consumers in green initiatives.

## Introduction

Environmental awareness has become a major concern for many consumers, and there is a growing preference for eco-friendly products and companies (Atkinson and Rosenthal, 2014; Kim et al., 2020; Zhuang et al., 2021). This is particularly relevant to the hospitality industry, as businesses like hotels consume large amounts of resources such as water and energy that negatively impact the environment (Jones et al., 2014). In response to consumer demand for sustainability, many hospitality businesses have adopted eco-friendly practices (Hsieh, 2012; Myung et al., 2012). For example, the hotel industry has embraced environmental sustainability to attract travelers who are seeking “green” brands (Lee, 2017). With 66% of millennials indicating that sustainability is an important factor in their hotel booking decisions (Trivago, 2019), the industry must adopt eco-friendly practices to stay competitive.

However, when the hospitality industry develops and communicates green initiatives, it often encounters consumer skepticism (Polonsky and Rosenberger, 2001; Matthes and Wonneberger, 2014), a response that aligns with predictions from attribution theory. Attribution theory, a psychological framework that examines how individuals interpret and ascribe causes to events or behaviors, can be employed to comprehend consumer skepticism toward the hospitality industry’s green initiatives (Kelley, 1973; Kelley and Michela, 1980).

This skepticism is fueled primarily by the perception that green initiatives, such as request to reuse towels, are motivated by cost savings (Goh and Balaji, 2016). Further, consumers may doubt the actual environmental impact of these initiatives, suspecting they merely serve as greenwashing tactics to attract environmentally conscious consumers without effecting real change (Szabo and Webster, 2021). Moreover, consumers may be skeptical about the motives of the hospitality industry as a whole, wondering if businesses are truly committed to sustainability or if they are simply paying lip service to the idea to appeal to the target market (Lee and Johnson, 2022; Hernandez et al., 2023).

To address these concerns and capitalize on consumer interest in eco-friendliness, hospitality companies must create effective green messaging strategies that not only promote their environmental practices but also appear authentic to the consumer. The primary purpose of this research is to propose and test two such message variables: message style (narrative vs. non-narrative) and message sidedness (two-sided vs. one-sided). These variables can lead to greater persuasiveness of green messages such as higher intent to participate in a green practice, by enhancing message usefulness and reducing skepticism.

Drawing on narrative persuasion theory, which posits that narrative messages featuring relatable characters and concrete details can lead to increased persuasion and reduced skepticism (Van Laer et al., 2014; Kim et al., 2021), we anticipate that a narrative message style will result in higher participation intent in a hotel’s green practices compared to a non-narrative style. Moreover, rather than relying exclusively on one-sided messages emphasizing only positive information, it is crucial to utilize two-sided messages that also disclose negative information. Such messages have been shown to be more effective in increasing persuasiveness, because they are perceived as more honest and resonate with consumers (Pechmann, 1992). This approach is particularly important in the hospitality industry, where consumer skepticism is already heightened, and therefore, balanced communication strategies are essential for fostering trust and promoting sustainable practices.

Overall, this research sheds light on the factors that increase the persuasiveness of green messages, offering valuable guidance for companies across various industries. By examining these factors, businesses not only in the hospitality sector but also in other sectors can benefit from this knowledge, allowing them to craft effective green messages that resonate with their target audiences and promote sustainable practices more successfully. Further, by addressing consumer skepticism and communicating eco-friendly initiatives in a way that is both useful and meaningful to consumers, companies can effectively connect and engage with their target audience, ultimately driving demand for their eco-friendly offerings. Given the growing trend of increasing consumer demand for sustainability and skepticism, the insights provided by this research are more valuable than ever.

## Literature review and hypotheses

### The effect of narrative (vs. non-narrative) message style on message effectiveness

Two common message styles are narrative and non-narrative messages. Narrative messages are presented in a story-like format, often featuring a testimonial, anecdote, or episodic memory (Escalas, 1998; Hinyard and Kreuter, 2007; Shen et al., 2015). Social media provides a unique space where narratives can take different forms, including blog updates, pictures, and Twitter feeds that provide a glimpse into the author’s ongoing personal narrative in life (Linde, 2015). Conversely, non-narrative messages may contain many forms, and are often presented as factual explanations or arguments, but they exclude the use of narratives (Padgett and Allen, 1997; Escalas, 2004).

Previous research suggests narratives are more effective than non-narratives by capturing attention, eliciting emotional responses, and connecting with the audience on a personal level (Chang, 2009; Escalas, 2012; Kim et al., 2017). Relatable characters and vivid details in narratives make the message more concrete, leading to increased message persuasion and reduced levels of skepticism (Escalas, 2004; Chang, 2009; Van Laer et al., 2014), particularly when testimonials or anecdotes based on real-life experiences are used (Dal Cin et al., 2004).

Moreover, narratives have the unique ability to transform abstract ideas into tangible, relatable concepts by incorporating temporal, physical, and social elements (Padgett and Allen, 1997; Green, 2006). The temporal structure imparts a sense of progression, illustrating how the abstract idea unfolds over time. Physical elements, such as setting and characters, situate the abstract idea within a specific place and time, making it more accessible and relatable. Social elements, including relationships and interactions between characters, demonstrate the impact of the abstract idea on people and their relationships, providing a context for its real-world implications.

Narratives effectively convey key concepts by grounding them in specific examples or experiences, rendering them more concrete and easier to comprehend (Escalas, 2004). By fostering a more comprehensive understanding of the underlying ideas, narratives facilitate consumer identification with the message, resulting in increased persuasion and engagement. Thus, narratives serve as a powerful tool for making abstract ideas tangible, relatable, and ultimately, more understandable.

Narrative messaging has the potential to overcome consumer skepticism about green marketing initiatives by providing useful information with vividness and details, while reducing potential skepticism. For example, a hotel may communicate its green initiatives in advertising and marketing venues through a short narrative that showcases the efforts of a couple who recently stayed at the hotel. The narrative could highlight how this couple contributed to the hotel’s green efforts, rather than simply presenting a list of facts. By displaying the couple along with their story, viewers can engage with the green messages and ultimately become less skeptical about the hotel’s green efforts by learning how the couple’s specific actions benefit the environment. Additionally, narrative green messaging has the potential to enhance consumers’ intention to participate in the hotel’s green efforts by making the message more relatable (Dessart and Pitardi, 2019). Therefore, by adopting a narrative approach, green marketing messages can effectively overcome consumer skepticism and promote consumer participation.

*H1*: Narrative (vs. non-narrative) style messages will result in greater perception of message usefulness (*H1*a), reduced skepticism (*H1*b), and higher intent to participate in the hotel’s green practice (*H1*c).

### The effect of one-sided (vs. two-sided) messages on message effectiveness

Consumers may exhibit skepticism toward advertisements that heavily emphasize environmental initiatives, as they may perceive these initiatives as opportunistic and driven by cost-saving concerns (Goh and Balaji, 2016). Attribution theory suggests that consumers may make extrinsic attributions about such initiatives, assuming that the hotel’s messaging is motivated by self-interest rather than genuine environmental concern (Ellen et al., 2000; Parguel et al., 2011). To overcome this skepticism, advertisers must devise strategies that present their environmental sustainability efforts in a more authentic and credible manner.

One approach to foster credibility is to incorporate a specific shortcoming or limitation, which can convey to the audience that the advertiser or brand is honest and trustworthy (Pechmann, 1992). Instead of solely emphasizing the positive aspects of a brand, research shows that a more effective approach is to present negative information alongside positive information, in what is referred to as a “two-sided message” (Rucker et al., 2008). This approach can encourage consumers to make an intrinsic attribution about a hotel’s messaging, leading to enhanced evaluations due to the perceived sincerity of the hotel’s motivations (Parguel et al., 2011).

Companies can enhance their credibility with skeptical consumers by acknowledging their product’s limitations or shortcomings, demonstrating transparency and honesty (Crowley and Hoyer, 1994; Winter and Krämer, 2012). A meta-analysis by O’Keefe (1999) supports the idea that two-sided messages are more effective than one-sided messages in changing attitudes and beliefs, particularly when the audience is aware of the opposing arguments, as explained by the reactance-based approach (Brehm and Brehm, 1981) and the counterargument availability-approach (Hovland et al., 1949). For instance, a hotel might promote its commitment to green practices (a positive attribute) while also acknowledging that it will financially benefit from those practices (a negative attribute).

Providing a more comprehensive and balanced view of the initiatives through two-sided messages can lead to increased trust and engagement from consumers, who appreciate transparency and honesty. Additionally, by offering a more complete picture of the green initiatives, including any potential self-serving attributes or limitations, consumers can make more informed decisions about whether or not to participate. Ultimately, incorporating a two-sided message strategy can help hotel brands better communicate the benefits and limitations of their green initiatives while building credibility and trust with consumers, leading to higher levels of participation in their green practices (Arbouw et al., 2019).

*H2*: Two-sided (vs. one-sided) messages will result in greater perception of message usefulness (*H2*a), less skepticism (*H2*b), and higher intent to participate in the hotel’s green practice (*H2*c) as compared to one-sided messages.

### The moderating effect of message sidedness

We posit the effectiveness of a narrative (vs. non-narrative) message style in encouraging consumers’ intent to participate in green practices can be further bolstered by using two-sided messages. This is because many consumers are skeptical of green claims and often distrust communication from companies (Calfee and Ringold, 1994; Obermiller and Spangenberg, 1998; Rahman et al., 2015). Two-sided messages have the potential to alleviate this skepticism by acknowledging unfavorable information about the brand itself, such as admitting financial gains from green practices. This approach can enhance the perceived utility, novelty, and honesty of the message (Kamins and Assael, 1987; Pechmann, 1992), and further enhance the already positive effect of a narrative message by equipping consumers with a more comprehensive understanding of the firm’s green initiatives. Further, voluntarily disclosing self-serving information is uncommon, and such disclosures are more likely to be seen as honest rather than opportunistic (Ellen et al., 2000; Parguel et al., 2011). Additionally, Forehand and Grier (2003) contend that public acknowledgement of the benefits that accrue to the company itself can inhibit the development of skepticism and negative reactions.

*H3*: Two-sided (vs. one-sided) green messages will enhance the effect of narrative (vs. non-narrative) style messages on perceived message usefulness (*H3*a) and skepticism (*H3*b).

### Downstream effect: serial mediator role of message usefulness and skepticism

As previously discussed, due to the prevalence of greenwashing in the advertising industry and the complexity and ambiguity of environmental issues (Lyon and Montgomery, 2013), the combination of narrative and two-sided messages can enhance the perceived usefulness of green messages by presenting more comprehensive and detailed information. We further anticipate that this heightened perceived usefulness will have a positive effect on consumers’ skepticism, ultimately leading to an increased intention to participate in green initiatives. When consumers perceive a message as useful and informative, they are more likely to consider it credible and trustworthy, which in turn mitigates their skepticism (Cheung et al., 2008). This argument is supported by a wealth of empirical evidence from the literature. For instance, prior research has demonstrated that the perceived usefulness of a message is positively associated with its perceived credibility (Kamins and Assael, 1987; DeLorme et al., 2009). Other studies have indicated that message usefulness can positively influence attitude and behavior change (Petty and Cacioppo, 1984; Alwitt and Prabhaker, 1994).

Skepticism has been identified as a crucial factor in determining the effectiveness of green advertising, as consumers tend to react negatively when they doubt the veracity of claims made (Obermiller et al., 2005; Yoo and MacInnis, 2005; Matthes and Wonneberger, 2014). This skepticism not only engenders adverse attitudes toward the message but also diminishes consumers’ willingness to engage in environmentally friendly behaviors, such as green purchases and participation in green programs (Forehand and Grier, 2003; Du et al., 2010; Yu et al., 2022). By positing a serial mediation effect involving message usefulness and skepticism, this research aspires to provide a more nuanced understanding of the combined influence of narrative message style and two-sided messaging on consumers’ involvement in green practices within the hospitality sector.

Top of Form

*H4*: The joint effects of message style and message sidedness on and participation intent in the hotel’s green practice will be serially mediated by message usefulness and skepticism.

## Method

Hypotheses were tested through a between-subjects experiment with a 2 × 2 design, varying message style (narrative vs. non-narrative) and message sidedness (two-sided vs. one-sided). A total of 260 participants were recruited from MTurk and compensated for their participation; all were at least 18 years old, US residents, and active on social media. Participants were diverse in age (*M* = 35.68, range 20–59), with 53% female, 72% white, and 53.5% college educated. On average, participants had three social media accounts, such as Facebook, Instagram, and Twitter.

*Stimuli.* The Facebook post stimuli were created with four different versions of the same message; the only differences were the manipulations used to achieve the desired effects (see Appendix A for complete details). The narrative version depicted a fictional couple who participated in the hotel’s green initiatives, while the non-narrative version simply stated that many guests of the hotel participated in the bed linens and towels reuse program. The two-sided message contained an acknowledgement of the hotel’s self-serving motive (i.e., money saving) for the green initiative. Facebook was chosen as the platform given its popularity among travel advertisers, with 79% of them using it for marketing purposes, and having the highest number of users compared to other social media platforms (Manoukian, 2019; Emarketer, 2022).

*Procedure.* Participants were informed of a United States based hotel chain’s upcoming social media campaigns on its new green initiatives and were asked for feedback. Only participants active on social media were included, and eligible participants were randomly assigned to one of four study conditions. They were instructed to view the stimuli in full-screen mode, and to respond to various dependent measures including measures of green participation intent, perceived message skepticism, message usefulness, environmental concern, manipulation checks for message style, and message sidedness, and demographic information. To reduce the likelihood of halo effects, a measure of green participation intent was collected prior to other variables. Additionally, the study included a distraction question asking participants to indicate how often they engaged in various leisure activities and a suspicion probe question asking participants to guess the objective and hypotheses of the study. Results indicate none of the participants guessed the hypotheses. To ensure participants were paying attention to the survey, we also included an attention check question asking how often they pay attention to TV commercials. We instructed participants to select “very frequently 7” as their response, regardless of their actual answer, to confirm that they were reading and following the instructions carefully. After eliminating 17 participants who failed the attention check, provided insincere responses, or were not native English speakers, a final dataset of 243 participants was obtained.

*Measures.* All of the variables were assessed on a seven-point Likert scale, ranging from “disagree” to “agree.” Green participation intent (α = 0.97, *M* = 4.93, *SD* = 1.42) was measured with two items taken from Rahman et al. (2015): “I have an intention to participate in the hotel’s green practice” and “I will participate in the hotel’s green practice.” Perceived green message skepticism (α = 0.97, *M* = 4.73, *SD* = 1.60) was measured with three items adopted from Mohr et al. (1998): “The green claims in the post I just saw are intended to mislead,” “I do not believe the hotel truly cares about the environment as it claims,” and “The green claims in the post are exaggerated.” Message usefulness (α = 0.97, *M* = 5.15, *SD* = 1.43) was measured with three items modified from Matthes and Wonneberger (2014): “I find information in the post useful,” “The information in the post is useful for my buying decisions,” and “The post delivers the information that I need for my buying decisions.” Environmental concern (α = 0.94, *M* = 4.90, *SD* = 1.71) was measured with three items taken from Schuhwerk and Lefkoff-Hagius (1995): “I am willing to make sacrifices to protect the environment,” “I am concerned about the environment,” and “The condition of the environment affects the quality of my life.” Message style manipulation was checked with three items (α = 0.95, *M* = 4.24, *SD* = 1.53) from Kim et al. (2017): “This message reads like a story,” This message shows characters engaged in specific actions to achieve goals,” and “This message mentions when, where, and how things happened.” Finally, message sidedness manipulation was checked with two items (*r* = 0.70, *M* = 4.17, *SD* = 1.47), “This message has both positive facts that help the hotel and negative facts that hurt the hotel” and “This message presents balanced information.” Descriptive statistics are shown in Table 1.

**Table 1:** Descriptive statistics.

Variable	*M*	*SD*	Min	Max
1. Message style	4.24	1.53	1	7
2. Message Sidedness	4.17	1.47	1	7
3. Message Usefulness	5.05	1.43	1	7
4. Skepticism	4.73	1.60	1	7
5. Green participation intent	4.93	1.42	1	7

## Results

*Randomization checks*. The four experiment conditions did not differ significantly in terms of participants’ gender (*χ*^2^ (3) = 0.48, *p* > 0.90), age (*F*(3,239) = 1.11, *p* = 0.35), education (*F*(3,239) = 1.44, *p* = 0.24), income (*F*(3,239) = 0.46, *p* = 0.70), or environmental concern (*F*(3,239) = 1.45, *p* = 0.23).

*Manipulation checks.* The manipulation check for message style was subjected to a 2 (message style: narrative vs. non-narrative) × 2 (message sidedness: two-sided vs. one-sided) ANOVA. First, the results confirmed a significant main effect of message style, (*F*(1,239) = 213.45, *p* < 0.001, *η*_p_^2^ = 0.47), indicating that participants perceived the narrative (*M* = 5.17, *SD* = 0.10) message as more story-like, action-oriented, and detailed compared to the non-narrative message (*M* = 3.06, *SD* = 0.10). The message sidedness manipulation also had a significant main effect (*F*(1,239) = 75.89, *p* < 0.001, *η*_p_^2^ = 0.24). Participants who viewed the two-sided message reported that the message contained more balanced information with both positive and negative aspects (*M* = 4.87, *SD* = 0.12) compared to the one-sided message (*M* = 3.43, *SD* = 0.12).

No significant two-way interactions were found for both cases. Therefore, manipulations were successful.

*Hypothesis tests. H1* through *H3* were examined with a series of 2 (message style: narrative vs. non-narrative) × 2 (message sidedness: two-sided vs. one-sided) ANOVAs. First, in support of *H1*a through *H1*c, the results confirmed a significant main effect of message style on message usefulness (*F*(1,239) = 74.41, *p* < 0.001, *η*_p_^2^ = 0.24), skepticism (*F*(1,239) = 79.52, *p* < 0.001, *η*_p_^2^ = 0.17), and green participation intent (*F*(1,239) = 55.80, *p* < 0.001, *η*_p_^2^ = 0.19). Specifically, the narrative messages resulted in higher message usefulness (*H1*a: *M_narrative_* = 5.77 vs. *M_non-narrative_* = 4.51), lower levels of skepticism (*H1*b: *M_narrative_* = 2.97 vs. *M_non-narrative_* = 4.10), and greater intent to participate in the hotel’s green practice (*H1*c: *M_narrative_* = 5.45 vs. *M_non-narrative_* = 4.28). Thus, *H1*a through *H1*c were supported.

For *H2*, the results verified a significant main effect of message style. As expected, the two-sided messages produced higher message usefulness (*H2*a: *F*(1,239) = 42.83, *p* < 0.001, *η*_p_^2^ = 0.15, *M_two-sided_* = 5.62 vs. *M_one-sided_* = 4.67), less skepticism (*H2*b: *F*(1,239) = 46.39, *p* < 0.001, *η*_p_^2^ = 0.16, *M_two-sided_* = 3.0 vs. *M_one-sided_* = 4.10), and greater intent to participate in the hotel’s green practice (*H2*c: *F*(1,239) = 34.80, *p* < 0.001, *η*_p_^2^ = 0.13, *M_two-sided_* = 5.32 vs. *M_one-sided_* = 4.04). Thus, *H2*a through *H2*c were supported.

For *H3*, we found a significant interaction between message style and message sidedness on message usefulness (*F*(1,293) = 10.30, *p* < 0.05, *η*_p_^2^ = 0.04) and skepticism (*F*(1,293) = 18.05, *p* < 0.001, *η*_p_^2^ = 0.007). Follow-up analyses were conducted within each level of message sidedness. Surprisingly, the difference between narrative and non-narrative messages was greater when the messages were one-sided (*M_narrative_* = 3.16 vs. *M_non-narrative_* = 3.81) rather than two-sided (*M_narrative_* = 2.76 vs. *M_non-narrative_* = 3.22). For skepticism, we found a similar pattern of results. The effect of narrative (vs. non-narrative) messages on skepticism was greater when the messages were one-sided (*M_narrative_* = 3.18 vs. *M_non-narrative_* = 5.02) rather than two-sided (*M_narrative_* = 2.76 vs. *M_non-narrative_* = 3.22). Thus, *H3*a and *H3*b were not supported.

**Figure 1:**
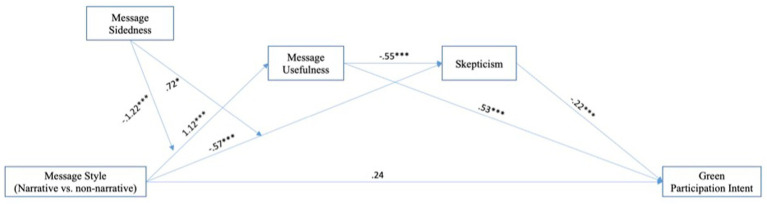
The moderated serial multiple mediation on green participation intent. *p < 0.05, **p < 0.01, ***p < 0.001.

*H4* was tested via a moderated serial mediator model using the bootstrapping procedure (10,000 samples) of the PROCESS” macro (model 84, Hayes, 2013) with message usefulness and skepticism as serial mediators. All variables that define indirect effects were mean-centered to minimize multicollinearity. As shown in Table 2 (refer to the last row), the index of moderated mediation result confirmed the joint effect of message style and message sidedness on intent to participate in the hotel’s green practice was serially mediated by message usefulness and skepticism (*B* = −0.15, 95% bias-corrected CI = −0.32 to −0.04). Interestingly, the results of the conditional indirect effect were consistent with *H3* findings, with the serial mediation effect found to be greater when the narrative messages were one-sided (*B* = 0.21, 95% bias-corrected CI = 0.06–0.41) compared to two-sided messages (*B* = 0.06, 95% bias-corrected CI = 0.01–0.14). As shown in Figure 1, the direct effect of message style was not significant in this model, indicating full mediation. Detailed path coefficients are shown Figure 1. Thus, *H4* was supported.

**Table 2:** Conditional indirect effects on green participation intent.

Indirect Path	Condition	*B*	*Boot SE*	Boot 95% CI
MS - skepticism - green participation intent	One-sided	0.20	0.09	0.04–0.41
	Two-sided	0.05	0.06	0.06–0.17
MS - MU - green participation intent	One-sided	0.51	0.20	0.56–1.34
	Two-sided	0.27	0.10	0.09–0.47
MS - MU- skepticism - green participation intent	One-sided	0.21	0.09	0.06–0.41
	Two-sided	0.06	0.03	0.01–0.14
Index of moderated serial mediation		−0.15	0.07	−0.32 to −0.04

MS, message style; MU, message usefulness.

## General discussion

While businesses are increasingly adopting environmentally-friendly practices to improve their public image, consumer skepticism toward green initiatives persists due to the perception that such efforts are mainly driven by self-interest (Matthes and Wonneberger, 2014). In this context, the findings of the present study have several important implications for both theory and practice.

First, this study highlights the importance of employing a narrative style as a method to augment the efficacy of green messages, offering novel insights into the manner in which message style influences consumer attitudes and behaviors. While extant literature has substantiated the persuasive power of narrative-based appeals across various contexts (Padgett and Allen, 1997; Escalas, 2004; Van Laer et al., 2014; Kim et al., 2017), the application of such approaches to message usefulness and green messaging has remained relatively unexplored. The current research advances the narrative persuasion literature by establishing a connection between the favorable impact of narratives and green communication, illustrating that the utilization of a narrative message style can enhance the perceived utility of messages while facilitating message processing.

The positive effect of the narrative message style can be attributed to its capacity to emotionally engage consumers, enhance message retention, facilitate comprehension, increase relatability, and mitigate skepticism (Escalas, 2004; Green, 2006; Chang, 2009). These factors collectively contribute to the persuasive power of narrative messages, rendering them particularly effective in addressing consumer skepticism toward green initiatives and promoting consumer participation (Van Laer et al., 2014; Kim et al., 2022). This finding implies that the adoption of narrative appeals can serve as an efficacious strategy in addressing consumer skepticism toward green initiatives, which is often engendered by concerns about a company’s self-interest (Matthes and Wonneberger, 2014).

Second, this study contributes to the extant literature by providing empirical evidence that two-sided green messages yield a significant positive effect on consumer responses. Attribution theory posits that consumers’ evaluations of green messaging are contingent upon their perceptions of the rationale behind the message’s creation (Kelley and Michela, 1980). Given the rising consumer skepticism that the hotel industry’s green initiatives already face, a hotel’s green messages may be perceived as opportunistic or manipulative (Forehand and Grier, 2003; Parguel et al., 2011). The inclusion of a self-serving motive in two-sided messaging can be construed as a comprehensive, candid, and transparent account of the hotel’s commitment to green initiatives. The positive impact of two-sided messages can be attributed to several factors, such as enhanced credibility (Pechmann, 1992; Crowley and Hoyer, 1994), increased message usefulness (DeLorme et al., 2009), reduced counterarguments (O’Keefe, 1999), and heightened message processing (Rucker et al., 2008). These factors contribute to a greater persuasive impact, beneficial not only in green marketing but also in other domains where trust and credibility are essential. By acknowledging potential profit motives, hotels can demonstrate their cognizance of consumer skepticism and their dedication to maintaining transparency and honesty in their communication. This approach, in turn, fosters trust and credibility with consumers, leading to higher levels of engagement and participation in the hotel’s green practices.

Third, regarding the moderating role of message sidedness, our initial hypothesis posited that two-sided messages would amplify the positive effect of narrative messages on message usefulness and attenuate skepticism. Contrary to our expectations, the findings revealed two-sided messages bolstered the impact of non-narrative messages more than narrative messages. A plausible explanation for this observation could be that non-narrative messages inherently possess weaker message usefulness and elicit heightened skepticism, rendering them more vulnerable to the influence of message sidedness. In contrast, narrative messages are already perceived as highly useful and evoke minimal skepticism, leaving scant scope for enhancement through the incorporation of message sidedness. Consequently, the enhanced effect of two-sided messages becomes more pronounced in the context of non-narrative messages. Further, our study makes a noteworthy contribution to scholarly discourse by proposing and empirically validating the serial mediation roles of message usefulness and skepticism in the combined effect of message style and message sidedness on intention to participate in green practices.

Regarding practical implications, our findings offer guidelines for companies seeking promote sustainable practices. First, results suggest adding a short narrative to the green message can significantly reduce consumer skepticism toward hotel’s sustainability efforts. This helps convey the hotel’s commitment and alleviate consumer doubts. However, authentic and ethical messaging is crucial to prevent unintended skepticism. Hotels’ sustainable practices. This is crucial because consumers are interested in sustainable hotels but skeptical of their motives. Incorporating a narrative can convey the authenticity of the hotel’s commitment and alleviate consumer skepticism. Second, this study highlights the importance of hotels being transparent about the self-serving benefits of their commitment to environmental causes. Openly acknowledging the financial gains of these initiatives can increase consumer trust and willingness to participate. Overall, our study suggests advertising focused on promoting green initiatives should consider using narrative and two-sided appeals, providing more information about specific actions and benefits of green practices. This can encourage consumer motivation to participate in sustainable practices.

### Limitations and future research

This study focused on social media as a message platform and did not explore the potential influence of others on the impact of green advertising. Future research could explore the impact of reference groups and social influence on consumers’ acceptance of green advertising and eco-tourism, in general. Such research could shed light on the role of social norms in shaping consumers’ attitudes and behaviors toward sustainability. Second, this study was conducted in the context of the hotel industry, and the findings may not be generalizable to other industries. Our interactions had small effect sizes and should be interpreted with caution, practically. Replication with larger samples may be useful. Future research could extend these findings to other industries such as fashion and consumer products, which are increasingly incorporating sustainability initiatives into their branding strategies. Nevertheless, this research provides important insight and implications for hotels seeking to utilize green message strategies.

## Data availability statement

The raw data supporting the conclusions of this article will be made available by the authors, without undue reservation.

## Ethics statement

The studies involving human participants were reviewed and approved by USC Institutional Review Board. The patients/participants provided their written informed consent to participate in this study.

## Author contributions

EKi: study conception and design, data collection and analysis, manuscript writing, project administration, and funding acquisition. EKw: manuscript writing. SH: manuscript writing and funding acquisition. HS: data anlaysis, study design, and manuscript writing. MS: manuscript writing and editing. All authors contributed to the article and approved the submitted version.

## Conflict of interest

The authors declare that the research was conducted in the absence of any commercial or financial relationships that could be construed as a potential conflict of interest.

## Publisher’s note

All claims expressed in this article are solely those of the authors and do not necessarily represent those of their affiliated organizations, or those of the publisher, the editors and the reviewers. Any product that may be evaluated in this article, or claim that may be made by its manufacturer, is not guaranteed or endorsed by the publisher.

## Supplementary material

The Supplementary material for this article can be found online at: https://www.frontiersin.org/articles/10.3389/fpsyg.2023.1176863/full#supplementary-material
