# Supplementary material for: Mollifying green skepticism: Effective strategies for inspiring green participation in the hospitality industry
Source: Front Psychol. 2023 Jun 15;14:1176863. doi: 10.3389/fpsyg.2023.1176863 (PMC10311023; doi:10.3389/fpsyg.2023.1176863)
Supplement: Supplementary file 1 [file Data_Sheet_1.pdf]

Appendix. Stimuli

Non-narrative/One-sided message

Hotel
1 min ·

The Little Things That Help Save Our Planet

Sustainability is integrated into everything we do. It's truly the mind behind our brand.

Last year, as part of our sustainability efforts, we launched new initiatives such as improved signage and enhanced information to encourage our guests to reuse their bed linens and towels.

Thanks to these new initiatives, last year many of our guests reused their bed linens and towels. Because of this, we were able to substantially reduce the huge amounts of laundry detergent and electricity needed to wash these items everyday.

We are very happy to do all we can to reduce our carbon footprint.

Picture of a Hotel Room

Narrative/One-sided message

Hotel
1 min ·

The Little Things That Help Save Our Planet

Sustainability is integrated into everything we do. It's truly the mind behind our brand.

Last year, as part of our sustainability efforts, we launched new initiatives such as improved signage and enhanced information to encourage our guests to reuse their bed linens and towels.

Thanks to these new initiatives, last year many of our guests reused their bed linens and towels. Because of this, we were able to substantially reduce the huge amounts of laundry detergent and electricity needed to wash these items everyday. Of course, we also saved a bunch of money in the process.

We are very happy to do all we can to reduce our carbon footprint.

Picture of a Hotel Room

** The image used was of a hotel bathroom vanity and sink. The bathroom was mostly smooth wood with some gray tile where the shower portion was slightly visible. There were folded towels under the sink and against the mirror. To the right of the sink was a small, wooden, shelf with a white orchid and a series of square, white, lit candles. Two additional bath towels were folded in cylinders under this ledge.

Non-narrative/Two-sided message

Hotel
1 min ·

The Little Things That Help Save Our Planet

Sustainability is integrated into everything we do. It's truly the mind behind our brand.

Last year, as part of our sustainability efforts, we launched new initiatives such as improved signage and enhanced information to encourage our guests to reuse their bed linens and towels.

Thanks to these new initiatives, Mr. and Mrs. Evans of Atlanta, GA, who stayed in our Los Angeles hotel last year, reused their bed linens and towels. Because of guests like Mr. and Mrs. Evans, we were able to substantially reduce the huge amounts of laundry detergent and electricity needed to wash these items everyday.

Picture of a Couple

We are very happy to do all we can to reduce our carbon footprint.

Narrative/Two-sided message

Hotel
1 min ·

The Little Things That Help Save Our Planet

Sustainability is integrated into everything we do. It's truly the mind behind our brand.

Last year, as part of our sustainability efforts, we launched new initiatives such as improved signage and enhanced information to encourage our guests to reuse their bed linens and towels.

Thanks to these new initiatives, Mr. and Mrs. Evans of Atlanta, GA, who stayed in our Los Angeles hotel last year, reused their bed linens and towels. Because of guests like Mr. and Mrs. Evans, we were able to substantially reduce the huge amounts of laundry detergent and electricity needed to wash these items everyday. Of course, we also saved a bunch of money in the process.

Picture of Hotel Room

We are very happy to do all we can to reduce our carbon footprint.

** The image used was of a smiling couple. A white man, in his 30s was wearing a gray, plaid shirt. His arm was around a white woman with light brown hair, in her 30s, wearing a blue, turtleneck, fleece shirt. The background was of a body of water surrounded by mountains.
